# Glutamate cycling may drive organic anion transport on the basal membrane of human placental syncytiotrophoblast

**DOI:** 10.1113/JP270743

**Published:** 2015-09-09

**Authors:** Emma M. Lofthouse, Suzanne Brooks, Jane K. Cleal, Mark A. Hanson, Kirsten R. Poore, Ita M. O'Kelly, Rohan M. Lewis

**Affiliations:** ^1^ University of Southampton, Faculty of MedicineSouthampton General HospitalTremona RoadSouthamptonUK

## Abstract

**Key points:**

- The placenta removes waste products, drugs and environmental toxins from the fetal circulation and two of the transport proteins responsible for this are OAT4 and OATP2B1 localised to the basal membrane of placental syncytiotrophoblast.
- We provide evidence that OAT4 and OATP2B1 mediate glutamate efflux when expressed in *Xenopus* oocytes and that in the perfused placenta, bromosulphothalein (an OAT4 and OATP2B1 substrate) stimulates glutamate efflux.
- Furthermore the efflux of glutamate can only be seen in the presence of aspartate, which will block glutamate reuptake by the placenta, consistent with cycling of glutamate across the basal membrane.
- We propose that glutamate efflux down its transmembrane gradient drives placental uptake via OAT4 and OATP2B1 from the fetal circulation and that reuptake of glutamate maintains this driving gradient.

**Abstract:**

The organic anion transporter OAT4 (SLC22A11) and organic anion transporting polypeptide OATP2B1 (SLCO2B1) are expressed in the basal membrane of the placental syncytiotrophoblast. These transporters mediate exchange whereby uptake of one organic anion is coupled to efflux of a counter‐ion. In placenta, these exchangers mediate placental uptake of substrates for oestrogen synthesis as well as clearing waste products and xenobiotics from the fetal circulation. However, the identity of the counter‐ion driving this transport in the placenta, and in other tissues, is unclear. While glutamate is not a known OAT4 or OATP2B1 substrate, we propose that its high intracellular concentration has the potential to drive accumulation of substrates from the fetal circulation. In the isolated perfused placenta, glutamate exchange was observed between the placenta and the fetal circulation. This exchange could not be explained by known glutamate exchangers. However, glutamate efflux was trans‐stimulated by an OAT4 and OATP2B1 substrate (bromosulphothalein). Exchange of glutamate for bromosulphothalein was only observed when glutamate reuptake was inhibited (by addition of aspartate). To determine if OAT4 and/or OATP2B1 mediate glutamate exchange, uptake and efflux of glutamate were investigated in *Xenopus laevis* oocytes. Our data demonstrate that in *Xenopus* oocytes expressing either OAT4 or OATP2B1 efflux of intracellular [^14^C]glutamate could be stimulated by conditions including extracellular glutamate (OAT4), estrone‐sulphate and bromosulphothalein (both OAT4 and OATP2B1) or pravastatin (OATP2B1). Cycling of glutamate across the placenta involving efflux via OAT4 and OATP2B1 and subsequent reuptake will drive placental uptake of organic anions from the fetal circulation.

**Abbreviations**
BM: basal membrane
BSP: bromosulphothalein
DHEAS: dehydroepiandrosterone‐3‐sulfate
OAT: organic anion transporter
OATP: organic anion transporting polypeptide
PAH: para‐aminohippuric acid

## Introduction

Many therapeutic drugs and environmental chemicals are potentially teratogenic in pregnancy. To protect the fetus, the placenta expresses transporters and enzymes that prevent transfer of xenobiotics to the fetus or remove them from the fetal circulation (Prouillac & Lecoeur, 2010; Staud & Ceckova, 2015). However, as the tragic example of thalidomide demonstrates, the placenta is by no means a perfect barrier.

The organic anion transporters (OATs) and the organic anion transporting polypeptides (OATPs) are two transporter families which mediate the transport of drugs and endogenous substrates in cells throughout the body (Hagenbuch & Stieger, 2013; Koepsell, 2013). In the human placenta, OAT4 (human gene symbol *SLC22A11*) and OATP2B1 (*SLCO2B1*) are localised to the fetal‐facing basal membrane (BM) and so will mediate transfer between the placenta and the fetal circulation (Ugele *et al*. 2003). The OATs and OATPs are widely expressed across species but OAT4 is found in humans and monkey but not in rodents (Koepsell, 2013). The OATs and OATPs are exchangers, mediating uptake of one molecule in exchange for efflux of another. In theory, this means that OAT4 and OATP2B1 could transport xenobiotics both to and from the fetal circulation. However, whether they transport xenobiotics in both directions or in only one direction will depend on the interaction with other substrates. For instance, the exchanger xCT (*SLC7A11*) can transport glutamate and cystine in either direction but in practice will primarily mediate cystine uptake. This is because the high intracellular glutamate concentration will competitively inhibit cystine efflux via xCT while driving uptake of cystine by secondary active transport (Sato *et al*. 1999).

Whether the OATs and OATPs on the BM of the placental syncytiotrophoblast transfer xenobiotics to the fetus, from the fetus or in both directions will depend on the balance of intra‐ and extracellular concentration of substrates. Intracellular glutamate concentrations in placenta, liver and muscle are reported to be greater than 4 mmol l^−1^ compared to 0.05 mmol l^−1^ in plasma, a transmembrane gradient of 80:1 (Philipps *et al*. 1978; Vinnars *et al*. 1980; Barle *et al*. 1996; Stanley, 2009). If OAT4 and OATP2B1 were coupled to an outwardly directed gradient such as glutamate this would drive uptake of xenobiotics out of the fetal circulation thus helping to protect the fetus. Furthermore, this would drive placental uptake of endogenous OAT substrates, including dehydroepiandrosterone‐3‐sulfate (DHEAS). The placenta cannot synthesise DHEAS and must take it up from the mother and the fetus in order to synthesise the oestrogen required to maintain pregnancy (Siiteri, 2005; Prouillac & Lecoeur, 2010). Coupling of OAT4 and OATP2B1 to an outwardly directed glutamate gradient would drive uptake of DHEAS and many xenobiotics from the fetal circulation and into the placenta. For this reason, it would make sense that these transporters were coupled to a counter‐ion; however, no known substrate of these transporters is present within the cell at comparable concentrations to glutamate. While OAT4 and OATP2B1 are not currently understood to transport glutamate, the fact that the related transporters, OAT2 and OAT10, do transport glutamate suggests that it is a possibility (Fork *et al*. 2011; Schulz *et al*. 2014).

Here we report glutamate exchange on the BM of human placental syncytiotrophoblast, which could not be explained by known transporters. We provide evidence that glutamate is a substrate for OAT4 and OATP2B1 and hence these transporters could mediate the observed glutamate exchange. We therefore propose that organic anion uptake from the feto‐placental circulation is coupled to glutamate cycling.

## Methods

Term placentas from uncomplicated pregnancies were collected with written informed consent from women delivering at the Princess Anne Hospital in Southampton with approval from the Southampton and Southwest Hampshire Local Ethics Committee (308/03/w). The study conformed to the *Declaration of Helsinki*.

### Placental perfusions

Placentas were perfused using the methodology of Schneider (Schneider *et al*. 1972) as adapted in our laboratory (Cleal *et al*. 2007, 2011). Placentas were perfused with Earle's bicarbonate buffer (EBB: 5 mmol l^−1^ glucose, 1.8 mmol l^−1^ CaCl_2_, 0.4 mmol l^−1^ MgSO_4_, 116.4 mmol l^−1^ NaCl, 5.4 mmol l^−1^ KCl, 26.2 mmol l^−1^ NaHCO_3_, 0.9 mmol l^−1^ NaH_2_PO_4_) and gassed with 5% CO_2_–95% O_2_ via the fetal catheter going into the chorionic plate fetal artery at 6 ml min^−1^ and via five maternal catheters at 14 ml min^−1^ using a roller pump.

Initial perfusions were performed with 58 nmol l^−1^ [^14^C]glutamate and 1.4 μmol l^−1^ [^3^H]proline (Perkin Elmer, MA, USA), which was perfused into the maternal artery. Proline was included to determine whether glutamate exchange could be mediated by the system ASC. To stimulate exchange, 16 μmol boluses of glutamate, glutamine, serine, salicylic acid, ibuprofen, para‐aminohippuric acid (PAH) and bromosulphothalein (BSP) were perfused into the fetal circulation.

A second set of perfusions were performed with 58 nmol l^−1^ [^14^C]glutamate (Perkin Elmer) which was perfused into the maternal artery. In these perfusions the first fetal arterial bolus was 100 μmol aspartate, the second 16 μmol BSP mixed with 100 μmol aspartate (as an inhibitor of glutamate reuptake by EAAT transporters, including SLC1A1, 2 and 3; Noorlander *et al*. 2004) and finally 16 μmol glutamate on its own. Boluses were injected in a volume of 1.5 ml EBB over a 10–15 s period.

When sampling, approximately 1.5 ml of venous exudate was collected from maternal and fetal venous outflows. Appearance of ^14^C‐tracer into the medium was measured by liquid scintillation counting (Packard‐Perkin Elmer, MA, USA). At the end of the experiments, the perfused mass of placental cotyledon (which assumed a white colour compared to non‐perfused tissue) was obtained by trimming off the non‐perfused tissue, blotting and weighing.

### Primary term human cytotrophoblast culture

Cytotrophoblast cells were isolated using an adaptation of the method developed by Kliman *et al*. (1986) as described previously (Greenwood *et al*. 1993). Isolated cells were plated in culture medium (Dulbecco's modified Eagle's medium and Ham's F‐12 1:1, 10% heat inactivated fetal calf serum, 0.6% glutamine and the antibiotics 1% gentamicin, 0.2% penicillin and 0.2% streptomycin) onto 35 mm culture dishes (Nunc), at a density of (2–2.5) × 10^6^, and were maintained in primary culture for 66 h at 37°C in a humidified incubator (95% air–5% CO_2_).

### rtPCR to determine exchanger gene expression in placenta

Total RNA was extracted from term human placental tissue or term human primary cytotrophoblast culture and reverse transcribed into cDNA. Primers were designed for *SLC7A13* (AGT‐1), the OATs and the OATPs (Table 1). rtPCR was carried out to determine expression under the following conditions: 94°C for 3 min; 40 cycles at 94°C for 30 s, 57–64°C (primer‐specific annealing temperatures are shown in Table 1) for 30 s and 72°C for 30 s; and then 72°C for 4 min. Positive results were confirmed by sequencing of the PCR product (GATC Biotech, Berlin, Germany). Where no expression was detected in placenta, primers were tested using cDNA from other cell lines or tissues thought to express the gene of interest as outlined in Table 1.

**Table 1:** **Primer and probe information**

Gene	Accession number	Forward primer (5′–3′)	Reverse primer (5′–3′)	Annealing temp. (°C)
*SLC7A13* (AGT‐1)	NM_138817.2	CTGTCCCAAAGCTGCCTAAG	GAATTTCCCTGGGTGTCAGA	60
*SLC22A6* (OAT1)	NM_153277.2	GTCTGCAGAAGGAGCTGACC	AGCAAGAGAGGTTCGGACAA	60
*SLC22A7* (OAT2)	NM_001184736.1	CCTCCAAGCTGCTGGTCTAC	CATCCCTGTCTGTCTGAGCA	60
*SLC22A8* (OAT3)	NM_018484.2	GTCCATACGCTGGTTGGTCT	GCTGTACCTCACCCGTGATT	60
*SLC22A11* (OAT4)	NM_018484.2	CAGAAAGGTGGCCAGGATAA	CACCAGCATGTTGGCTAGAA	60
*SLC22A10* (OAT5)	NM_001039752.3	CACAGAACCCTGTGTGGATG	TCGGAAGACATAAGCCAAGC	60
*SLC22A9* (OAT7)	NM_080866.2	AGGTTTGGGAGAAGGTTCGT	TGCTGCCTCCAGTTCTTTTT	60
*SLCO1A2* (OATP1A2)	NM_001145211.2	GCTTGTCTTGCTGGTTGTGA	GAATCCATTAAAGCGCCAAA	60
*SLCO1B3* (OATP1B3)	NM_019844.3	GTGGCTTGGTTTCCTTGTGT	AGTTGCAACCGTAGGAATGG	60
*SLCO2A1* (OATP2A1)	NM_005630.2	ACGGTTTCCATGCATCTTTC	ATGGCAAGGGGAGAGGTACT	60
*SLCO2B1* (OATP2B1)	NM_001145211.2	GGGAACACAGCCTTGATTGT	CCATCATGGTCACTGCAAAC	60
*SLCO3A1* (OATP3A1)	NM_013272.3	TGCGGTGCCTTACTCTTCTT	TAGCAAGCAGTGGACACCAG	60
*SLCO4A1* (OATP4A1)	NM_013272.3	CCCGTCTACATTGCCATCTT	CTCAGGCTGAACTGGGACTC	61
*SLCO4C1* (OATP4C1)	NM_180991.4	GAGAAGCTCCGGTCACTGTC	TGGGCACAGAATCATCAAGA	60

### cRNA synthesis for microinjection into oocytes

Plasmids containing the cDNA of human OAT4 or human OATP2B1 obtained from OriGene (OriGene Technologies Inc., Rockville, MD, USA) were linearised using restriction enzymes (Promega, Southampton, UK). Both plasmids contained two T7 promoter sites and were double digested before purification to prevent synthesis of cRNA from the non‐coding T7 site. The OAT4 plasmid was digested with the restriction enzymes *Nde*I and *Stu*I. The OATP2B1 plasmid was digested with the restriction enzymes *Nde*I and *Xho*I. cRNA was synthesised from the coding T7 promoter using the Ambion mMachine mMessage kit according to the manufacturer's instructions (Life Technologies, UK).

### *Xenopus* oocyte trans‐stimulation studies

*Xenopus laevis* oocytes were obtained from the European *Xenopus* Resource Centre (Portsmouth, UK). Oocytes were treated with collagenase (2 mg ml^−1^) in buffer OR2 (2.5 mmol l^−1^ KCl, 82.5 mmol l^−1^ NaCl, 1 mmol l^−1^ CaCl_2_, 1 mmol l^−1^ Na_2_HPO_4_, 1 mmol l^−1^ MgCl_2_, 5 mmol l^−1^ Hepes) for 1 h at room temperature. Oocytes were then incubated in ND91 buffer (2 mmol l^−1^ KCl, 91 mmol l^−1^ NaCl, 1.8 mmol l^−1^ CaCl_2_, 1 mmol l^−1^ MgCl_2_, 5 mmol l^−1^ Hepes, 1% penicillin/streptomycin and 0.1% gentamycin sulphate) overnight at 18°C. Stage V oocytes were injected with 20 ng of the cRNA for the transporter of interest dissolved in 56 nl of water. The water‐injected control oocytes were injected with an equivalent volume of water.

Two to three days after injection of the relevant transporter cRNA, oocytes were preloaded with [^14^C]glutamate by incubating for 30 min in 20 μmol l^−1^ (50 μCi l^−1^) [^14^C]glutamate in ND91 buffer and then washed 3 times in 1 ml of ND91 buffer to remove extracellular label. Initial time course experiments were performed to show the [^14^C]glutamate efflux at 2, 5 and 8 min. For OAT4 time courses, oocytes were trans‐stimulated by addition of 10 mmol l^−1^ glutamate to the extracellular ND91 buffer. For OATP2B1 time courses, oocytes were trans‐stimulated by addition of 20 mmol l^−1^ BSP to the extracellular ND91 buffer. For OAT4, ND91 control experiments were only performed at 5 min. Extracellular BSP was used to trans‐stimulate glutamate efflux in OATP2B1 expressing oocytes as in preliminary experiments glutamate was not shown to trans‐stimulate significant glutamate efflux.

Based on time course experiments, further experiments were performed at 5 min. For OAT4 the substrates added to extracellular ND91 buffer to trans‐stimulate efflux were 10 mmol l^−1^ glycine, 10 mmol l^−1^ aspartate, 10 mmol l^−1^ para‐aminohippuric acid (PAH), 10 mmol l^−1^ ibuprofen, 10 mmol l^−1^ glutamate, 10 and 20 mmol l^−1^ estrone sulphate (E‐3‐S) and 10 and 20 mmol l^−1^ BSP. For OATP2B1, the substrates added to extracellular ND91 buffer to trans‐stimulate efflux were 10 and 20 mmol l^−1^ glycine, 10 mmol l^−1^ glutamate, 10 and 20 mmol l^−1^ E‐3‐S, 10 and 20 mmol l^−1^ BSP and 5 mmol l^−1^ pravastatin. For each condition, efflux was determined in three batches of 10 oocytes injected with the cRNA of interest and three batches of 10 control oocytes not expressing the transporter of interest. Efflux of ^14^C‐tracer into the medium was measured by liquid scintillation counting (Packard‐Perkin Elmer).

Each independent experiment used oocytes from a different frog and experiments were conducted in different weeks.

### Data analysis and statistics

For the perfusion experiments, trans‐stimulation of glutamate release was determined by calculating the area under the curve (AUC) for the 15 min following injection using the trapezium rule with the three points before the bolus (*t* = −1, −2 and −3 min) and the points following (*t* = 15, 17 and 21 min) defined as baseline. To determine whether the AUC was greater than 0 (indicating efflux) the data were analysed using Wilcoxon's signed rank test.

For the oocyte experiments, total [^14^C]glutamate efflux for each substrate over 5 min was compared to that of ND91 buffer alone using Dunnett's two‐tailed multiple comparison's *t* test. Significance was assumed at *P* < 0.05 and data are presented as means ± SEM. For the time course experiments, [^14^C]glutamate efflux for each substrate and buffer alone was determined at 2, 5 and 8 min and was compared to that of ND91, and a regression line was plotted for efflux against time. Analysis was performed using IBM SPSS Statistics version 21.

## Results

### Glutamate stimulates release of [^14^C]glutamate into the fetal circulation of the perfused placenta, while other known OAT and OATP substrates do not

To identify the transporters mediating glutamate exchange on the BM of the placental syncytiotrophoblast, substrates of known amino acid exchangers were added to the fetal circulation of the isolated perfused placenta to determine if they would stimulate glutamate efflux.

Average perfused cotyledon weight was 45.5 ± 3.5 g and average fetal venous recovery was 5.0 ± 0.2 ml min^−1^.

**Figure 1:**
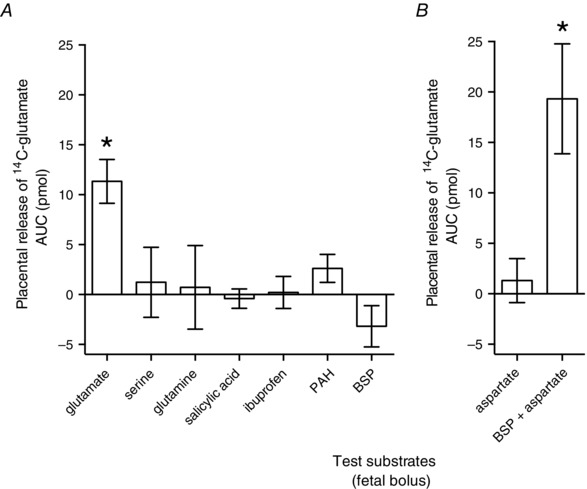
**Trans‐stimulation of [^14^C]glutamate efflux into the fetal circulation of the isolated perfused placenta following addition of boluses of potential exchanger substrates to the fetal circulation** *A*, trans‐stimulation of [^14^C]glutamate efflux into the fetal circulation was observed in exchange for fetal glutamate, but none of the other potential exchange substrates. Data presented represent the increase in glutamate transfer as the area under the curve (AUC) above baseline release. *P = 0.002 *vs*. baseline, *n* = 3–12. *B*, aspartate bolus alone did not stimulate glutamate efflux but aspartate in combination with the OAT4 and OATP2B1 substrate BSP (used in combination to inhibit glutamate reuptake by EAAT transporters) stimulated observable exchange. *n* = 5, *P = 0.04 *vs*. baseline.

Over the course of the experiments, the baseline percentage of the [^14^C]glutamate and [^3^H]proline perfused into the maternal circulation reaching the fetal circulation was 1.7 ± 0.3% for [^14^C]glutamate and 3.6 ± 0.6% for [^3^H]proline. Addition of a 16 μmol glutamate bolus to the fetal circulation stimulated significant release of [^14^C]glutamate (above baseline transfer) into the fetal circulation (*P* = 0.002, *n* = 12, Fig. 1 *A*). No release of [^14^C]glutamate above baseline was observed following 16 μmol fetal boluses of serine (*n* = 5 placentas), glutamine (*n* = 5 placentas), salicylic acid (*n* = 5 placentas), or BSP (*n* = 3 placentas, Fig. 1 *A*). Proline transfer was not altered following any of the fetal boluses (data not shown).

In the second set of perfusions there was no release of glutamate above baseline following an aspartate bolus (*n* = 4, *P* = 0.76), but BSP + aspartate (*n* = 5, *P* = 0.04) stimulated release of [^14^C]glutamate from the placenta (Fig. 1 *B*).

### Expression profile of OAT/OATP in term human placenta and primary cytotrophoblast culture

**Figure 2:**
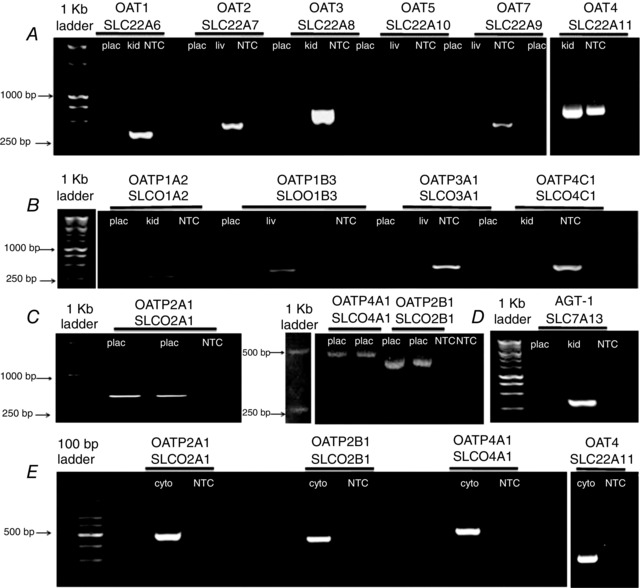
**Expression of OATs and OATPs in term human placenta and primary cytotrophoblast** *A*, rtPCR amplification of cDNA from term human placenta, control tissue (kidney or liver) and no template control (NTC) for the OATs. *B*, rtPCR amplification of cDNA from term human placenta, control tissue (kidney, brain or liver) and NTC for OATP1A2, OATP1B3, OATP3A1, OATP4C1. *C*, rtPCR amplification of cDNA from term human placenta and NTC for OATP2A1, OATP4A1 and OATP2B1. *D*, rtPCR amplification of cDNA from term human placenta, control tissue (kidney) and NTC for AGT‐1. *E*, rtPCR amplification of cDNA from primary term human cytotrophoblast culture and in NTC for OATP2B1, OATP2B1, OATP4A1 and OAT4. plac, placenta; kid, kidney; liv, liver; cyto, cytotrophoblast.

To confirm reports in the literature that the known glutamate exchangers AGT‐1 and OAT2 were not expressed in the placenta (Matsuo *et al*. 2002; Fork *et al*. 2011), rtPCR was performed on placental cDNA. rtPCR was performed to confirm the presence of mRNA for key members of the OAT and OATP transporter families in placenta and cytotrophoblast culture. Expression of SLC22A11 (OAT4), SLCO2A1 (OATP2A1), SLCO2B1 (OATP2B1) and SLCO4A1 (OATP4A1) were detected in placenta and primary cytotrophoblast culture (Fig. 2), and the result was confirmed by sequencing of the PCR product. No expression of SLC22A6 (OAT1), SLC22A7 (OAT2), SLC22A8 (OAT3), SLC22A10 (OAT5), SLC22A9 (OAT7), SLCO1A2 (OATP1A2), SLCO1B3 (OATP1B3), SLCO3A1 (OATP3A1), or SLCO4C1 (OATP4C1) was detected in placenta or primary trophoblast cDNA although the primers did amplify product in control tissues (Fig. 2). No expression of the glutamate–aspartate exchanger *SLC7A13* (AGT‐1) mRNA was detected in human placenta by rtPCR (Fig. 2).

### Glutamate efflux from oocytes expressing OAT4 or OATP2B1

In order to test the hypothesis that OAT4 and OATP2B1 transported glutamate, these proteins were expressed in *Xenopus* oocytes and transport studies undertaken.

To account for endogenous glutamate release, release of [^14^C]glutamate efflux was measured from oocytes not expressing the protein of interest and this efflux was subtracted from the release measured from oocytes expressing the protein of interest. After preloading by incubating with 20 μmol l^−1^ [^14^C]glutamate for 30 min, there was no significant difference in glutamate uptake in the water‐injected (17.3 ± 1.7 pmol/oocyte, *n* = 7), non‐injected (19.5 ± 2.0 pmol/oocyte, *n* = 10), OAT4‐injected (20.6 ± 2.6 pmol/oocyte, *n* = 10) or OATP2B1‐injected (17.3 ± 1.6 pmol/oocyte, *n* = 5) oocytes. Assuming an intra‐oocyte volume of 0.4 μl (Taylor & Smith, 1987), this is the equivalent of 48 μmol l^−1^ [^14^C]glutamate in a total intracellular glutamate concentration of 4 mmol l^−1^, indicating that there will be significant tracer dilution within the oocyte.

**Figure 3:**
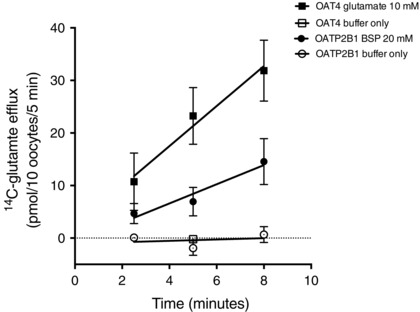
**Trans‐stimulation of glutamate efflux by extracellular exchange substrates increases in a linear manner between 2 and 8 min** In *Xenopus* oocytes expressing OAT4 or OATP2B1 the addition of extracellular exchange substrate (glutamate for OAT4 and BSP for OATP2B1) led to a time‐dependent increase in [^14^C]glutamate efflux. In contrast, there was no efflux in the absence of extracellular exchange substrate. Data are means and SEM, *n* = 3.

When trans‐stimulated with 10 mmol l^−1^ glutamate, efflux of [^14^C]glutamate from OAT4‐injected oocytes increased in a linear manner with time (*R* = 0.73, *n* = 3, Fig. 3). Similarly, efflux of [^14^C]glutamate from OATP2B1‐injected oocytes increased in a linear manner with time when trans‐stimulated with 20 mmol l^−1^ BSP (*R* = 0.67, *n* = 3, Fig. 3).

### Trans‐stimulation of glutamate efflux from *Xenopus* oocytes expressing OAT4

**Figure 4:**
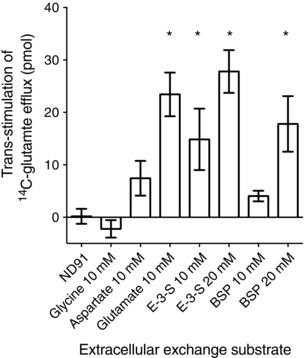
**Trans‐stimulation of [^14^C]glutamate from *Xenopus* oocytes expressing OAT4** Efflux of intracellular [^14^C]glutamate was trans‐stimulated by the extracellular exchange substrates glutamate, estrone‐sulphate (E‐3‐S) and bromosulphothalein (BSP). *P < 0.05 *vs*. ND91, *n* = 5 independent experiments. Data represent total efflux of [^14^C]glutamate into buffer in 5 min.

The function of OAT4 when expressed in oocytes was tested by measuring the ability of known OAT4 substrates to trans‐stimulate [^14^C]glutamate efflux. In oocytes expressing OAT4, there was significant OAT4 mediated efflux of [^14^C]glutamate following a 5 min incubation with 10 mmol l^−1^ glutamate (*n* = 10), 10 mmol l^−1^ (*n* = 5) or 20 mmol l^−1^ (*n* = 5) estrone sulphate or 20 mmol l^−1^ BSP (*n* = 5) (*P* < 0.05, Fig. 4) when compared to control oocytes. Importantly, there was no significant efflux of [^14^C]glutamate from OAT4 expressing oocytes following a 5 min incubation with ND91 buffer alone (*n* = 10) or with 10 mmol l^−1^ glycine (*n* = 8), 10 mmol l^−1^ BSP, or 10 mmol l^−1^ aspartate (*n* = 5) (Fig. 4).

### Trans‐stimulation of glutamate efflux from *Xenopus* oocytes expressing OATP2B1

**Figure 5:**
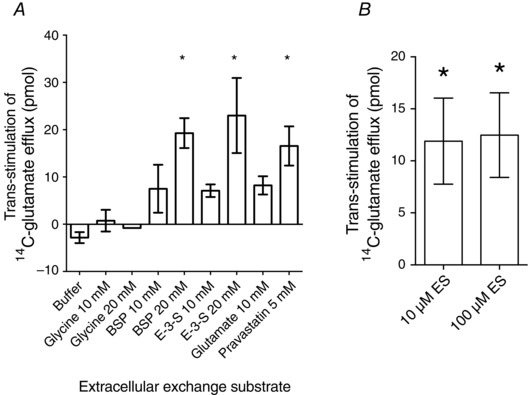
**Trans‐stimulation of [^14^C]glutamate from *Xenopus* oocytes expressing OATP2B1** Efflux of intracellular [^14^C]glutamate was trans‐stimulated by the extracellular exchange substrates 5 mmol l^−1^ pravastatin, 20 mmol l^−1^ estrone‐sulphate (E‐3‐S) and 20 mmol l^−1^ bromosulphothalein (BSP). *P < 0.05 *vs*. ND91 Buffer, *n* = 5 independent experiments. Data represent total efflux of [^14^C]glutamate into buffer in 5 min.

The function of OATP2B1 when expressed in oocytes was tested by measuring the ability of known OATP2B1 substrates to trans‐stimulate [^14^C]glutamate efflux. In oocytes expressing OATP2B1, when compared to control oocytes, there was significant OATP2B1 mediated efflux of [^14^C]glutamate following a 5 min incubation with 20 mmol l^−1^ estrone‐3‐sulphate (*n* = 5), 20 mmol l^−1^ BSP (*n* = 5) and 5 mmol l^−1^ pravastatin (*n* = 3) (*P* < 0.05, Fig. 5). There was no significant efflux of [^14^C]glutamate from OATP2B1 expressing oocytes following a 5 min incubation with ND91 buffer alone (*n* = 5), 10 or 20 mmol l^−1^ glycine (*n* = 5), 10 mmol l^−1^ glutamate (*n* = 5), 10 mmol l^−1^ estrone sulphate or 10 mmol l^−1^ BSP (Fig. 5). OATP2B1 therefore appears to mediate glutamate efflux but not glutamate uptake.

## Discussion

This study demonstrates for the first time that OAT4 and OATP2B1 mediate glutamate efflux and that, in the placenta, uptake of the OAT4 and OATP2B1 substrate BSP is coupled to release of glutamate. That glutamate can act as a counter‐ion for these transporters is physiologically important, as its high intracellular concentration will provide a chemical gradient to drive placental uptake of OAT4 and OATP2B1 substrates from the fetal circulation. Furthermore, rapid reuptake of glutamate released by OAT4 and OATP2B1 will maintain this gradient. These findings suggest that organic anion transport is coupled to cycling of glutamate across the BM of placental syncytiotrophoblast.

The initial perfusion data demonstrate exchange of glutamate for glutamate but not for other substrates of any candidate transporters including OAT4 and OATP2B1. To explain these observations we proposed that there was glutamate efflux by exchange, but that the [^14^C]glutamate released from the placenta did not appear in the fetal vein due to rapid reuptake by the high affinity EAAT transporters on the BM (Day *et al*. 2013). In the one case where [^14^C]glutamate efflux was observed, in exchange for a fetal glutamate bolus, this can be explained by the fact that the fetal glutamate bolus will both stimulate [^14^C]glutamate efflux and competitively inhibit its reuptake by the EAAT transporters. Consistent with our new proposal, while there was no exchange for aspartate alone, in the presence of aspartate the high affinity OAT4 and OATP2B1 substrate BSP did stimulate observable release of [^14^C]glutamate.

Consistent with previous studies we show expression OAT4, OATP2B1, OATP2A1 and OATP4A1 in the human placenta (St‐Pierre *et al*. 2002; Ugele *et al*. 2003). However, no other OAT or OATP transporters were shown to be expressed. In addition, we confirm that the glutamate exchanger *SLC7A13* (AGT‐1) is not expressed in human placenta and so cannot be mediating the observed glutamate exchange (Matsuo *et al*. 2002).

Glutamate uptake by the syncytiotrophoblast BM is known to be mediated by highly accumulative EAAT transporters (Day *et al*. 2013). The fact that efflux of [^14^C]glutamate could only be observed in the presence of glutamate or aspartate, which would competitively inhibit these transporters, suggests that they also mediate rapid uptake of glutamate released by OAT4 and or OATP2B1. Glutamate cycling on the BM of the placental syncytiotrophoblast would energetically couple organic anion transport to the Na^+^ gradient maintained by the Na^+^/K^+^‐ATPase. Recycling glutamate will maintain the intracellular glutamate gradient and thus the driving force for organic anion uptake. EAAT transporters are widely expressed and glutamate cycling could potentially drive influx of OAT4 and OATP2B1 substrates in other cell types. Glutamate concentrations in fetal plasma are around 36 μmol l^−1^ (Cetin *et al*. 2005) suggesting that there may be futile cycling of glutamate via OAT4. Exchangers which use the gradients of higher abundance substrates to accumulate lower abundance substrates will inevitably undertake futile cycling to some degree. While it may appear inefficient, futile cycling does not alter the transmembrane gradient and is energetically neutral so it does not matter to the cell as long as the lower abundance (but probably higher affinity) substrates are accumulated within the cell. However, as the EAAT transporters in the BM have a high affinity for glutamate (Kanai *et al*. 2013), it is likely that glutamate concentrations at the basal membrane are lower than in the fetal vein and futile cycling is minimised.

**Figure 6:**
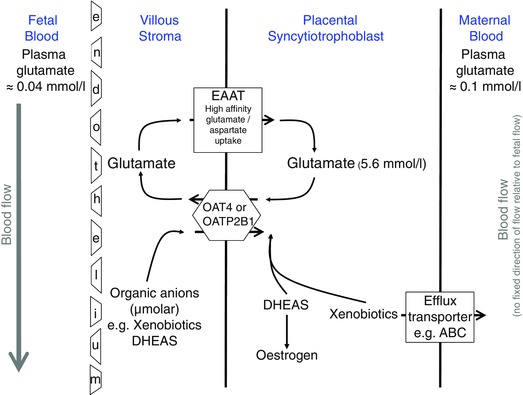
**The glutamate gradient may drive uptake of OAT4 and OATP2B1 substrates from the fetus** Once in the placenta these can be transported to the mother in the case of xenobiotics or, in the case of DHEAS, used for placental synthesis of oestrogen. Glutamate reuptake is mediated by high affinity EAAT glutamate transporters which maintain the glutamate gradient. Glutamate efflux in exchange for OAT substrates could only be observed in the perfused placenta if reuptake via EAAT was blocked with aspartate. This suggests recycling of glutamate back to the syncytiotrophoblast.

Both OAT4 and OATP2B1 are expressed in the placenta and have been localised to the BM (Ugele *et al*. 2003). While neither of these transporters had previously been shown to transport glutamate, the observation of [^14^C]glutamate efflux in exchange for their substrate BSP suggested that this was a possibility. When expressed in *Xenopus* oocytes, both OAT4 and OATP2B1 were indeed found to mediate glutamate efflux. OAT4 was also found to mediate glutamate uptake and so is most likely to be responsible for the observed glutamate for glutamate exchange. In the placenta, coupling BM organic anion transport to the glutamate gradient would provide a strong driving force for the removal of xenobiotics and uptake of steroid sulphates from the fetal circulation (Fig. 6). This is because high glutamate levels will competitively inhibit efflux of other substrates and increase the rate at which the binding site is recycled to the extracellular face of the membrane. Both inhibiting efflux and increased opportunity for uptake will increase the accumulation of extracellular substrates within the cell. OAT4 and OATP2B1 are also expressed in other tissues, such as kidney and liver, where the glutamate gradient would enhance their ability to drive intracellular accumulation of biological or pharmacological substrates (e.g. statins) (Hagenbuch & Stieger, 2013; Koepsell, 2013).

Previous studies have suggested that α‐ketoglutarate and orotic acid may provide the countervailing gradients for specific OATs (Pritchard, 1995; Fork *et al*. 2011), yet the transmembrane gradient of these substrates is low compared to that of glutamate, limiting their potential to drive accumulation of extracellular substrates (Panitchob *et al*. 2015). However, the relative affinities of the potential exchange substrates, such as α‐ketoglutarate, may also need to be considered. That transport by OATP2B1 is coupled to a significant substrate gradient is consistent with the observation that the anti‐diabetic drug glibenclamide, a substrate of OATP2B1, has an intracellular concentration 50 times that in plasma, something which would require an active transport mechanism (Hagenbuch & Stieger, 2013; Day *et al*. 2013).

There are several experimental considerations which need to be taken into account when interpreting these data. First, that [^14^C]glutamate taken up into the placenta or oocyte will become diluted in a much larger pool of endogenous glutamate. This means that when measuring efflux we will only measure a small proportion of the glutamate coming out (proportional to the dilution of [^14^C]glutamate in the intracellular pool). This could explain why relatively high levels of exchange substrate were required to see maximal efflux. Secondly we cannot exclude conversion of [^14^C]glutamate to α‐[^14^C]ketoglutarate. As α‐ketoglutarate, and other anionic metabolites of glutamate, are potentially substrates of these transporters, we cannot exclude that we are measuring efflux of these rather than glutamate. However, given the dilution of tracer within the cell, production of α‐ketoglutarate will primarily occur from unlabelled glutamate and as such, any α‐[^14^C]ketoglutarate would be highly dilute. Furthermore, aspartate would not be expected to block reuptake of α‐[^14^C]ketoglutarate. These issues could be addressed in future experiments using liquid chromatography–mass spectrometry (LCMS) based approaches.

Glutamate was able to trans‐stimulate efflux of intracellular [^14^C]glutamate via OAT4, but we did not see clear evidence for trans‐stimulation of glutamate efflux via OATP2B1. This provides indirect evidence that glutamate is taken up by OAT4 but as we did not observe uptake directly we cannot exclude the possibility that glutamate was acting as a trans‐activator, as has been observed to occur for benzoic acid and OAT2 (Pfennig *et al*. 2013). Direct uptake studies are also required to determine whether or not OATP2B1 mediates glutamate uptake. Differential intra‐ and extracellular substrate affinity has previously been observed for other exchangers including the recent observation that OAT10 mediates glutamate efflux but not influx (Meier *et al*. 2002; Schulz *et al*. 2014). OATP transporters, including OATP2B1, have been shown to have higher and lower affinity binding sites and this needs to be taken into account in future kinetic analysis of glutamate uptake and efflux by OATP2B1 and OAT4 (Shirasaka *et al*. 2012; Liu *et al*. 2013).

While the focus of this work was on the placenta, the findings are potentially relevant to all cells expressing these transporters. In the case of OATP2B1, the glutamate gradient would increase its ability to accumulate its substrates, e.g. statins, within hepatocytes where they can inhibit 3‐hydroxy‐3‐methylglutaryl‐coenzyme A (HMG‐CoA) and in muscle where they are linked to statin‐induced myopathy (Hagenbuch & Stieger, 2013). OAT4 is expressed on the apical membrane of the renal proximal tubular epithelium where it has been hypothesised to mediate reuptake of organic anions in exchange for an unidentified intracellular anion which we suggest is likely to be glutamate (Ekaratanawong *et al*. 2004).

The observation that glutamate efflux occurs by OAT2, OAT4, OAT10 and OATP2B1 raises the possibility that it is a more widespread feature of the OAT and OATP families than had previously been appreciated (Fork *et al*. 2011; Schulz *et al*. 2014). This family characteristic may have eluded detection as most studies measure uptake, but if, as for OATP2B1, glutamate efflux was the predominant mode of action, studies focusing on uptake may not have observed glutamate transport.

Understanding the gradients that determine the activity of these exchange transporters will also be important for pharmacokinetic modelling of drug transport in the placenta and other tissues (Lewis *et al*. 2013; Staud & Ceckova, 2015). Modelling also provides a framework within which to test our assumptions about the nature of transporter activity (Panitchob *et al*. 2015; Widdows *et al*. 2015).

This study demonstrates that OAT4 and OATP2B1 mediate glutamate efflux and that the uptake of organic anions across the BM of placental syncytiotrophoblast may be coupled to cycling of glutamate. Coupling organic anion transport to glutamate cycling would mean that they are able to accumulate substrates within the cell much more effectively than if exchanging for a counter‐ion with a lower transmembrane gradient. These findings have implications for our understanding of the biology and pharmacokinetics of OAT4 and OATP2B1 (Watanabe *et al*. 2011). Further studies in other members of the OAT and OATP families are warranted to determine if glutamate cycling is a driver of their activity.

## Additional information

### Competing interests

The authors have no competing interests to declare.

### Author contributions

R.L., E.L., S.B., K.P., M.H. and I.O. were involved in conception and design of experiments. E.L. undertook all experimental analysis. E.L., R.L., K.P. and I.O. were involved in data analysis. All authors contributed to the writing of the paper and approved the final version for publication.

### Funding

This work was funded by the Gerald Kerkut Charitable Trust and the BBSRC (BB/L020823/1, BB/I011315/1). M.A.H. was funded by the British Heart Foundation (CH‐02‐01).
